# Supplementary material for: Exploring the relationship between local food environments and obesity in UK, Ireland, Australia and New Zealand: a systematic review protocol
Source: BMJ Open. 2018 Feb 22;8(2):e018701. doi: 10.1136/bmjopen-2017-018701 (PMC5855298; doi:10.1136/bmjopen-2017-018701)
Supplement: Supplementary file 1 [file bmjopen-2017-018701supp001.pdf]

Supplementary APPENDIX

Search strategy developed for MEDLINE (OVID)

1. Food environment*.tw
2. Fodscape*.tw
3. Food access.tw
4. Food availability.tw
5. Food affordability.tw
6. Food outlet access.tw
7. Food price.tw
8. Food store*.tw
9. Food outlet*.tw
10. Grocer*.tw
11. Convenience store*.tw
12. Supermarket*.tw
13. Restaurant*.tw
14. Food bank*.tw
15. Online shopping.tw
16. Food shopping.tw
17. Economic access.tw
18. Residential.tw
19. Neighbo*.tw
20. Neighbourhood.tw
21. Urban area*.tw
22. Obes*.tw
23. Obesogenic.tw
24. Overnutrition/
25. Overnutrition.tw
26. Over nutrition.tw
27. Overweight/
28. Body Mass Index/
29. BMI.tw
30. Weight adj2 change.tw.
31. Weight status.tw

32. Weight control.tw
33. Waist circumference.tw
34. OR/1-21
35. OR/22-33
36. 34 AND 35
