# Supplementary material for: Exploring the relationship between local food environments and obesity in UK, Ireland, Australia and New Zealand: a systematic review protocol
Source: BMJ Open. 2018 Feb 22;8(2):e018701. doi: 10.1136/bmjopen-2017-018701 (PMC5855298; doi:10.1136/bmjopen-2017-018701)
Supplement: Supplementary file 2 [file bmjopen-2017-018701supp002.pdf]

Preliminary data extraction form

**<u>Reviewer identification</u>**<br>Date:<br>Reference ID:<br>Name:
**<u>Article description</u>**<br>Title:<br>Authors:<br>Institution:<br>Corresponding email:<br>Type of source (journal article/ grey literature):<br>Year:
**<u>Aims and study design</u>**<br>Aims/objectives:<br>Study design:
**<u>Methodology:</u>**<br>1. Settings<br>a) Country/city:<br>b) Urban/rural area:<br>2. Special characteristics of the population:<br>a) Age structure:<br>b) Sex structure:<br>c) Ethnic features:<br>d) Educational level:<br>e) Socioeconomic level:<br>f) Other characteristics:

3. Study exclusions:<br><br>4. Exposure:<br><br>a) Food environment definition:<br><br>b) Food sources/outlet definition:<br><br>c) Data source<br><br>d) Year exposure data collected:<br><br>e) Type of food environment variable:<br><br>f) Food environment variable definition:<br><br>g) Methods of data collection<br><br>5. Outcomes:<br><br>a) Reported outcomes:<br><br>b) Outcomes definition:<br><br>c) Type of measure (self-reported/measured):
**Statistical Analysis**<br><br>a) Modelling strategy<br>b) Reported bias<br>c) Reported confounders
**Key findings:**<br><br>Main findings:
**Limitations:**<br><br>Study limitations:
