# Supplementary material for: Exploring the relationship between local food environments and obesity in UK, Ireland, Australia and New Zealand: a systematic review protocol
Source: BMJ Open. 2018 Feb 22;8(2):e018701. doi: 10.1136/bmjopen-2017-018701 (PMC5855298; doi:10.1136/bmjopen-2017-018701)
Supplement: Author's manuscript [file bmjopen-2017-018701.draft_revisions.pdf]

BMJ Open is committed to open peer review. As part of this commitment we make the peer review history of every article we publish publicly available.

When an article is published we post the peer reviewers' comments and the authors' responses online. We also post the versions of the paper that were used during peer review. These are the versions that the peer review comments apply to.

The versions of the paper that follow are the versions that were submitted during the peer review process. They are not the versions of record or the final published versions. They should not be cited or distributed as the published version of this manuscript.

BMJ Open is an open access journal and the full, final, typeset and author-corrected version of record of the manuscript is available on our site with no access controls, subscription charges or pay-per-view fees (http://bmjopen.bmj.com).

If you have any questions on BMJ Open's open peer review process please email editorial.bmjopen@bmj.com

BMJ Open

Exploring the relationship between local food environments and obesity in UK, Ireland, Australia and New Zealand: A systematic review protocol

Journal:	*BMJ Open*
Manuscript ID	bmjopen-2017-018701
Article Type:	Protocol
Date Submitted by the Author:	25-Jul-2017
Complete List of Authors:	Fuentes Pacheco, Andrea; University of Edinburgh School of Molecular Genetic and Population Health Sciences, Centre of Population Health Sciences, Usher Institute of Population Health Sciences and Informatics<br>Carrillo Balam, Gabriela; University of Edinburgh School of Molecular Genetic and Population Health Sciences, Centre of Population Health Sciences, Usher Institute of Population Health Sciences and Informatics<br>Archibald, Daryll; University of Edinburgh, Scottish Collaboration for Public Health Research & Policy, Usher Institute of Population Health Sciences and Informatics; La Trobe University College of Science Health and Engineering, School of Psychology and Public Health<br>Grant, Elizabeth; University of Edinburgh School of Molecular Genetic and Population Health Sciences, Global Health Academy, Usher Institute of Population Health Sciences<br>Skafida, Valeria; University of Edinburgh, School of Social and Political Science
Keywords:	obesity, food environments, neighbourhood, food access, systematic review

SCHOLARONE™
Manuscripts

Exploring the relationship between local food environments and obesity in UK, Ireland, Australia and New Zealand: A systematic review protocol

Andrea Fuentes Pacheco[1], Gabriela Carrillo Balam[1], Daryll Archibald[2,3], Liz Grant[4], Valeria Skafida[5]

[1]Centre of Population Health Sciences, Usher Institute of Population Health Sciences and Informatics, The University of Edinburgh, Edinburgh, UK.

[2]Scottish Collaboration for Public Health Research & Policy, Usher Institute of Population Health Sciences and Informatics, The University of Edinburgh, Edinburgh, UK

[3]School of Psychology and Public Health, College of Science, Health and Engineering, La Trobe University, Bundoora, Melbourne

[4]Global Health Academy, Usher Institute of Population Health Sciences and Informatics, The University of Edinburgh, Edinburgh, UK

[5]School of Social and Political Science, University of Edinburgh, UK.

Corresponding author details

Andrea Fuentes Pacheco

Centre of Population Health Sciences

Usher Institute of Population Health Sciences and Informatics

The University of Edinburgh

Room no. 815, Doorway 3, Old Medical School

For peer review only

Teviot Place, Edinburgh EH8 9AG

United Kingdom

E-mail: Andrea.Fuentes@ed.ac.uk

Tel: +44(0)131 650 3214

Keywords: obesity, food environments, neighbourhoods, deprivation, food access, food availability, healthy food, unhealthy food, systematic review

Word count: 2376

For peer review only

For peer review only - http://bmjopen.bmj.com/site/about/guidelines.xhtml

ABSTRACT

Introduction: Obesity is a global pandemic that affects all socioeconomic segments, however, the worst figures have been observed in the most disadvantaged social groups [1, 2]. Evidence from US and Canada have shown that specific urban settings like deprived neighbourhoods encourage an "obesogenic" behaviour in the population living and/or working there [2-5]. We aim to examine the evidence related to the association between local food environments and obesity in UK, Australia and New Zealand, identify gaps in the evidence related to this particular association and analyze the pertinence of this relationship considering the regional context.

Methods: six platforms from 1990 to 2017 will be searched: Medline (Ovid), EMBASE (Ovid), Scopus, CINAHL, ASSIA and Web of Science. Grey literature will be also searched on Opengrey Europe (SIGLE), The Grey Literature Report and relevant governmental sites related to the countries included on this review. Additional studies will be retrieved from the reference lists of the selected articles. It will be included cohort, longitudinal, case - study and cross-sectional studies that have assessed the relationship between food environments and obesity in UK, Ireland, Australia and New Zealand regardless the sex, age and ethnicity of the population. Two researchers will independently select the studies and extract the data. Data items will include: authors´ names, study name, study design, year of study, year exposure data collected, country, city, urban/rural, age range, study exclusions, special characteristics of study population, aims, working definitions of food environments and food outlets, methods of data collection, outcomes reported and key findings. A narrative synthesis and a summary of the results separately for children and adults will be performed, according to the type of food exposure – outcome. All the selected studies will be assessed using The Effective Public Health Practice Project (EPHPP) tool [6].

For peer review only - http://bmjopen.bmj.com/site/about/guidelines.xhtml

Ethics and dissemination: due to this study is based on published literature there is no need for Ethics approval. Our findings will be presented in pertinent national and international scientific conferences and publish in a peer –reviewed journal.

Strengths and limitations of this study

- This is the first systematic review that will geographically extend the work done by Cobb et al. [4], analysing the evidence about the relationship between food environments and obesity in UK, Ireland, Australia and New Zealand.
- This study also will incorporate the economic access variable as part of the food environment and an additional analysis of the quality of foodstuff (healthy and unhealthy) available inside the residential areas measured.
- It will be undertaken a comparative analysis between the review results and the North American findings. This is highly relevant in order to contextualize if this phenomenon respond to a regional influence or if this is occurring in other zones of the world
- Despite we cannot cover the whole European and Oceanic region we chose these high – income countries from each region to conduct this review and compare the results with the North American findings later in the discussion stage. The UK, Ireland, Australia and New Zealand are among the countries with the highest figures of obesity in Western Europe and Oceania [7]. These countries are also experiencing a post nutritional transition with a strong influence of a globalized and industrialized food market that lead us to believe obesogenic food environments could be present.

For peer review only - http://bmjopen.bmj.com/site/about/guidelines.xhtml

BACKGROUND

The obesity pandemic has been increasing dramatically on a global scale since 1980 [8, 9]. According to the World Health Organization (WHO), over 600 million people worldwide were obese in 2014 [9]. The increased trend has particularly affected high and upper middle - income countries that have high industrialization and urbanisation. However, a growing number of low and middle income countries are also dealing with this pandemic and the additional burden of malnutrition [1, 10]. Whilst the prevalence is rising across all the population segments, the worst figures have been observed in the most disadvantaged social groups [3, 6].

This condition, regarded in some literature as a disease [11] is described as one of the most complex health problems of modernity due to its multi causal etiology [1, 2]. Beyond individual determinants, it has been proposed that there is a strong influence of the complex social and physical contexts in which individual behavioural decisions are made [12]. Based on this approach and focusing on obesity, Swinburn and Egger defined the concept of food environment as the "sum of the influences that the surroundings, opportunities or conditions of life have on promoting obesity in individuals and populations" [13]**.** Many studies have shown that specific urban settings like deprived neighbourhoods encourage an "obesogenic" behaviour in the population living and/or working there [2-5]. In those areas, food environments act to enhance excessive energy intake and weight gain in the medium and long term [3, 5].

Food environments involve access and availability to (and quality of) foodstuffs [9]. Access is divided into physical and economic access. Physical access refers to the distance and number of food sources like food retail stores, restaurants and takeaways from the centroids of spatial units of analysis, commonly represented by residential areas. Economic access is

For peer review only - http://bmjopen.bmj.com/site/about/guidelines.xhtml

mediated by the residents' food purchase capacity and retail food prices [8]. The availability depends on the variety of food provision which is directly related with the quality, which is most frequently divided into healthy and unhealthy [13]. Thus, in essence, local access and availability to both nutritious and unhealthy food in neighbourhoods, are considered a crucial part of the obesogenic environment [14]. These variables have a direct influence on food shopping behavior and eating patterns, ultimately affecting the nutritional status in the population that live and / or work within those areas [2, 5, 14, 15].

Despite this, there is still a major need of clarification with regard to the many pathways involved in this obesogenic influence [2, 5]. It has been suggested that a lack of daily physical access to large stores combined with less availability, and higher price of healthy products could discourage the daily consumption of these type of products. The aforementioned, foster a more frequent intake of cheaper and more palatable food provided generously by small and nearby stores [2, 14, 16]. The excessively unhealthy food on offer inside food stores increases the consumption of a wide range of highly caloric and less nutritive processed products [14, 16]. This food pattern could lead to a rapid increase in body fat over time, leading to overweight and, obesity [2, 14]. A considerable volume of evidence has been generated in North America on this subject during the last decade. According to Cobb et al., in spite of many studies from US and Canada found a null or negative relationship, a substantial number of them also showed a positive association between the different food environment variables and the prevalence of obesity among children and adults [4]. It is highlighted among the findings, for example, the strong relationship between the higher fast food availability inside stores and fast food outlets density with obesity among the residents of those areas [4].

In the case of European and Oceanic countries, there is a lack of updated evidence pertaining to the association between local food environments and obesity. The UK, Ireland, Australia

For peer review only - http://bmjopen.bmj.com/site/about/guidelines.xhtml

and New Zealand are among the countries with the highest figures of obesity in Western Europe and Oceania [7]. After USA, Mexico and Chile these 4 countries are leading the obesity ranking in the OECD group [7]. In addition, the figures also reflect that the prevalence is more concentrated in disadvantaged areas [6, 9]. As with the US and Canada, these countries are also experiencing a post nutritional transition with a strong influence of a globalized and industrialized food market that lead us to believe obesogenic food environments could be present [6, 9]. Considering the previous, it was selected these high – income countries from each region to conduct this systematic review and compare the results with the North American findings later in the discussion stage.

Until now, three systematic reviews and one scoping review have been conducted, focusing on the US and Canada only [4, 15, 17, 18]. The first study analyzed the whole physical environments, including transportation and physical activity access [15]. The second was limited to the consumer retail food environments without the relationship with obesity and only the third embraced a closer review question but again, considering that geographic scope [4, 18]. This review plans to extend that work geographically, whilst also incorporating a focus on economic access [13, 14] as part of food environment, which has not been undertaken in previous reviews. This is the first systematic review that will attempt to cover more variables within a food environment, considering the following dimensions: availability of foodstuffs inside food stores and economic and physical food access. An additional analysis of each variable according the quality of foodstuff (healthy and unhealthy) to which people are exposed in residential zones will be carried out in the studies coming from in UK, Ireland, Australia and New Zealand.

Cummins and Macintyre (2006) recommend a regional analysis of the available research in other high - income countries in order to identify if the social, economic and geographic contexts are similar enough to attribute the same causes at global scale [12]. Although

For peer review only - http://bmjopen.bmj.com/site/about/guidelines.xhtml

neighbourhood differences in obesity are noticeable in many countries, the most consistent evidence for a "contextual" effect of food environment is really only evident in North America [12]. This would need further explanation or contextualization as to its relevance. Furthermore, following a variety of public health and private interventions created to promote healthy lifestyle within neighbourhoods, such as the insertion of a higher number of supermarkets in deprived areas and social media campaigns about healthy eating [19], it will be useful to find out if this phenomenon is affecting the most vulnerable population living in these European and Oceanic countries.

Objectives

The primary objective is to examine the evidence related to the association between local food environments and obesity in UK, Australia and New Zealand. The specific objectives are to identify gaps in the evidence related to this particular association and analyze the pertinence of this relationship considering the regional contexts.

METHODS

The methods used in this review will draw on the methodology developed by Cobb et al. in 2015 who explored the relationship between local food environments and obesity in US and Canada [4]. The present work will extend the geographic scope of that work by focusing on studies conducted in UK, Ireland, Australia and New Zealand. It will mantain the physical food access and availability dimensions included in the review by Cobb et al. and will incorporate a third dimension: economic access. Finally, it will be also guided by The Preferred Reporting Items for Systematic Reviews and Meta-Analysis Protocols (PRISMA - P) [20].

For peer review only - http://bmjopen.bmj.com/site/about/guidelines.xhtml

Eligibility criteria

Types of studies

It will be included observational epidemiological studies that have assessed access and/or availability of food sources inside neighbourhoods (cohort, longitudinal, case - study and cross-sectional) with group level data and with individual level data on more than 200 people. It will be excluded the studies with less than 200 people. This sample size cut - point was proposed by Cobb et al. considering that studies with a smaller sample would be statistically underpowered for the detection of a significant association between the variables [4, 6]. For the purposes of this study it will be followed the same criterion due to we would like to compare both reviews in a later discussion.

Participants

Eligible participants will include human population regardless of age, sex or ethnicity. It will be excluded individuals with major pathologies, pregnant women, homeless population, breastfeeding women and participants with major physical limitations due to all the conditions aforementioned could independently alter the nutritional status. Individuals with obesity grade 3 will also be excluded because this is the most severe stage of the obesity and according to the evidence [20], there are another biological causes involved in that status (increases in morbid obesity).

Years considered

It will be included studies from January 1990 and May 2017.

Setting

The sample will include research articles only from UK, Ireland, Australia and New Zealand.

For peer review only - http://bmjopen.bmj.com/site/about/guidelines.xhtml
For peer review only

Language

Only articles written in English due to is the predominant language of the selected countries.

Information sources

Database searches and other sources to identify studies

It will be searched the following databases: MEDLINE (Ovid), EMBASE (Ovid), Scopus, CINAHL, ASSIA and Web of Science. Grey literature will be searched on Opengrey Europe (SIGLE), The Grey Literature Report, relevant governmental sites related to the countries included on this review (UK Foresight programme, Australian Institute of Health and Welfare, National library of Australia, PANDORA (Australian Government Web Archive) and Obesity Policy Coalition Australia) and other international organizations websites related to this topic (World Obesity Federation, Spotlight project and European Association of the Study of Obesity (EASO). Additionally, the reference lists of the selected articles will be checked for additional articles that can potentially be retrieved.

Search strategy

For peer review only

For peer review only - http://bmjopen.bmj.com/site/about/guidelines.xhtml

The first step involved a preliminary scan in Medline with the purpose to identify and build a list of index and free terms (see supplementary Appendix). The final list of search terms was consulted with a senior librarian at The University of Edinburgh and with the review team. However, due to the iterative nature of the search process, additional search terms and sources may be incorporated into the search strategy.

Study records

Data management

Retrieved studies from databases, grey literature and hand – searching will be exported to Endnote Library. The programme will be also used for the screening and deduplication process.

Selection process

Two researchers (AF and GC) will independently undertake the studies selection and data extraction and any discrepancies will be solved by consensus between the two authors. If required, a third party (DA) will make a judgment on the data entered and act as an arbitrator.

Data extraction

Data will be extracted into Excel using a customised data extraction form which will be updated during the review process. The reviewers will develop the extraction separately**.**

Data items

Features to be extracted will include:

- Author´s names

For peer review only - http://bmjopen.bmj.com/site/about/guidelines.xhtml

For peer review only

- Design: study name, study design, year of study, year exposure data collected, country, city, urban/rural, age range, study exclusions, special characteristics of study population
- Aims and working definitions of food environments and food outlets
- Methods of data collection
- Outcomes reported: outcome definition, self – reported or measured
- Key findings

Outcomes and prioritisation

The primary outcome is obesity. The diagnosis of this pathology has to follow the criterion established by the World Health Organization to classify the obesity with a Body Mass Index (BMI) over 30 kg/m^2 [9]. Change in BMI as well as measurements of weight and height will be accepted in order to have the option to calculate and classify the nutritional status.

The secondary outcome is central obesity, represented by waist circumference and waist to hip ratio (only if the primary outcome will not be included).

Risk of bias in individual studies

All the selected studies will be assessed using The Effective Public Health Practice Project (EPHPP) tool [6]. The ítems contained are:

- Selection bias
- Study design
- Confounders
- Blinding
- Data collection methods

For peer review only - http://bmjopen.bmj.com/site/about/guidelines.xhtml

- Withdrawals and drop-outs
- Intervention integrity
- Analyses

Data synthesis

A narrative synthesis and a summary of the results separately for children and adults will be performed, according to the type of food exposure – outcome. The measurements techniques chosen in every study will be also analyzed. Finally, it will be compared the main findings with those ones generated in the original American systematic review

Subgroups analysis: men, women, low SES, high SES, urban rural

Protocol registration

A detailed protocol was registered with the International Prospective Register of Systematic Reviews (PROSPERO): 2017:CRD42017068193 (http://www.crd.york.ac.uk/PROSPERO/display_record.asp?ID=CRD42017068193). The study will be guided by the Preferred Reporting Items for Systematic review and Meta - Analysis Protocols (PRISMA-P) 2015 statement (20).

CONCLUSIONS

In the past years, other systematic reviews have assessed the evidence related to the association between food environments and obesity, focusing mainly on the studies coming from US and Canada and representing the food environment exposure through the measurement of physical food access and food availability inside neighbourhoods. This

For peer review only - http://bmjopen.bmj.com/site/about/guidelines.xhtml

systematic review is the first study that will geographically extend the work done by Cobb et al., analyzing the evidence about this relationship in UK, Ireland, Australia and New Zealand. Furthermore, it will incorporate the economic access variable as part of the food environment and an additional analysis of the quality of foodstuff (healthy and unhealthy) available inside the residential areas measured. Finally, it will undertake a comparative analysis between the review results and the North American findings. This is highly relevant in order to contextualize if this phenomenon respond to a regional influence or if this is occurring at global scale.

ETHICS AND DISSEMINATION

There is no need for Ethics approval due to this study is based on published literature. However, this work is subject to Institutional Review Board oversight by The University of Edinburgh's Centre for Population Health Sciences. This work is however subject to Institutional Review Board oversight by The University of Edinburgh's Centre for Population Health Sciences. The review findings will be presented at scientific conferences and be published in the peer-reviewed scientific journal

Competing interests

None declared.

Funding

DA is supported jointly by the Medical Research Council (MRC; MR/K023209/1) and the Chief Scientist Office (CSO).

Contributors

Andrea Fuentes conceived the idea for this work and drafted the protocol under the supervision of Daryll Archibald, Liz Grant and Valeria Skafida. Gabriela Carrillo suggested

For peer review only - http://bmjopen.bmj.com/site/about/guidelines.xhtml

valuable corrections to the manuscript. All the authors will be involved in the systematic review process

REFERENCES

1. Popkin BM. The Nutrition Transition: An Overview of World Patterns of Change. Nutr Rev. 2004;62(7):S140-S3.

2. Foresight Programme. Tackling Obesities - Future Choices – Project Report. Government Office for Science, 2010.

3. Lake A, Townshend T. Obesogenic environments: exploring the built and food environments. J R Soc Promot Health. 2006;126(6):262-7.

4. Cobb L, Appel L, Franco M, et al. The relationship of the local food environment with obesity: A systematic review of methods, study quality and results. Obesity (Silver Spring). 2015;23(7):1331–44.

5. Foresight Programme. Tackling Obesities: Future Choices – Obesogenic Environments – Evidence Review. Government Office for Science, 2007.

6. Effective Public Health Research Project. Qualitiy Assessment Tool for Quantitative Studies (EPHPP). McMaster Evidence-Based Practice Centre, 2010.

For peer review only - http://bmjopen.bmj.com/site/about/guidelines.xhtml

7. OECD. Obesity and the Economics of Prevention: Fit not Fat, OECD Publishing; 2010.

8. World Health Organization. Global Atlas on cardiovascular disease, prevention and control. Mendis S, Puska P, Norrving B editors, 2011.

9. World Health Organization. Fact sheets: Obesity and overweight, 2016.

10. Popkin BM, Adair L, Wen Ng S. NOW AND THEN: The Global Nutrition Transition: The Pandemic of Obesity in Developing Countries. Nutr Rev. 2012;70(1):3–21.

11. Jensen MD, et al. AHA/ACC/TOS Guideline for the Management of Overweight and Obesity in Adults: a report of the American College of Cardiology/American Heart Association Task Force on Practice Guidelines and The Obesity Society. American Heart Association, 2013.

12. Cummins S, Mcintyre S. Food environments and obesity - neighbourhood or nation? Int J Epidemiol. 2006;35:100-4.

13. Swinburn B, Egger G, Raza F. Dissecting Obesogenic Environments: The Development and Application of a Framework for Identifying and Prioritizing Environmental Interventions for Obesity. Prev Med 1999;29((6 Pt 1)):563-70.

14. Drewnowski A. Obesity, diets, and social inequalities. Nutr Rev. 2009;67(Suppl. 1):S36–S9.

15. Mackenbach JD, Rutter H, Compernolle S, et al. Obesogenic environments: a systematic review of the association between the physical environment and adult weight status, the SPOTLIGHT project. BMC Public Health. 2014;14:233.

16. Franch C, Hernandez P, Weisstaub G, et al. Consideraciones para identificar barreras y potencialidades culturales para el control y prevención del sobrepeso y obesidad en mujeres de bajos recursos. Fondo Nacional de Desarrollo e Investigación en Salud (FONIS), 2012.

17. Minaker LM, Shuh A, Olstad DL, et al. Retail food environments research in Canada: A scoping review. Can J Public Health. 2016;107(0):5344.

18. Ni Mhurchu C, Vandevijvere S, Waterlander W, et al. Monitoring the availability of healthy and unhealthy foods and non-alcoholic beverages in community and consumer retail food environments globally. Obes rev. 2013;14(1):108–19.

19. Scottish Public Health Network. Obesity Route Map. 1.0 ed: Scottish Government; 2011.

20. Shamseer L, Moher D, Clarke M, et al. PRISMA-P Group. Preferred reporting items for systematic review and meta-analysis protocols (PRISMA-P) 2015: elaboration and explanation. BMJ. 2015;2(349):g7647.

For peer review only - http://bmjopen.bmj.com/site/about/guidelines.xhtml

For peer review only

For peer review only - http://bmjopen.bmj.com/site/about/guidelines.xhtml

Supplementary APPENDIX

Search strategy developed for MEDLINE (OVID)

1. Food environment*.tw
2. Fodscape*.tw
3. Food access.tw
4. Food availability.tw
5. Food affordability.tw
6. Food outlet access.tw
7. Food price.tw
8. Food store*.tw
9. Food outlet*.tw
10. Grocer*.tw
11. Convenience store*.tw
12. Supermarket*.tw
13. Restaurant*.tw
14. Food bank*.tw
15. Online shopping.tw
16. Food shopping.tw
17. Economic access.tw
18. Residential.tw
19. Neighbo*.tw
20. Neighbourhood.tw
21. Urban area*.tw
22. Obes*.tw
23. Obesogenic.tw
24. Overnutrition/
25. Overnutrition.tw
26. Over nutrition.tw
27. Overweight/
28. Body Mass Index/
29. BMI.tw
30. Weight adj2 change.tw.
31. Weight status.tw

For peer review only

32. Weight control.tw
33. Waist circumference.tw
34. OR/1-21
35. OR/22-33
36. 34 AND 35

For peer review only

PRISMA-P (Preferred Reporting Items for Systematic review and Meta-Analysis Protocols) 2015 checklist: recommended items to address in a systematic review protocol*

Section and topic	Item No	Checklist item	
**ADMINISTRATIVE INFORMATION**			
Title:			
Identification	1a	Identify the report as a protocol of a systematic review	1
Update	1b	If the protocol is for an update of a previous systematic review, identify as such	NA
Registration	2	If registered, provide the name of the registry (such as PROSPERO) and registration number	13
Authors:			
Contact	3a	Provide name, institutional affiliation, e-mail address of all protocol authors; provide physical mailing address of corresponding author	1
Contributions	3b	Describe contributions of protocol authors and identify the guarantor of the review	13
Amendments	4	If the protocol represents an amendment of a previously completed or published protocol, identify as such and list changes; otherwise, state plan for documenting important protocol amendments	NA
Support:			
Sources	5a	Indicate sources of financial or other support for the review	14
Sponsor	5b	Provide name for the review funder and/or sponsor	14
Role of sponsor or funder	5c	Describe roles of funder(s), sponsor(s), and/or institution(s), if any, in developing the protocol	NA
**INTRODUCTION**			
Rationale	6	Describe the rationale for the review in the context of what is already known	5-8
Objectives	7	Provide an explicit statement of the question(s) the review will address with reference to participants, interventions, comparators, and outcomes (PICO)	8
**METHODS**			
Eligibility criteria	8	Specify the study characteristics (such as PICO, study design, setting, time frame) and report characteristics (such as years considered, language, publication status) to be used as criteria for eligibility for the review	8-9
Information sources	9	Describe all intended information sources (such as electronic databases, contact with study authors, trial registers or other grey literature sources) with planned dates of coverage	10
Search strategy	10	Present draft of search strategy to be used for at least one electronic database, including planned limits, such that it could be repeated	10
Study records:			
Data management	11a	Describe the mechanism(s) that will be used to manage records and data throughout the review	10

For peer review only

Selection process	11b	State the process that will be used for selecting studies (such as two independent reviewers) through each phase of the review (that is, screening, eligibility and inclusion in meta-analysis)	11
Data collection process	11c	Describe planned method of extracting data from reports (such as piloting forms, done independently, in duplicate), any processes for obtaining and confirming data from investigators	11
Data items	12	List and define all variables for which data will be sought (such as PICO items, funding sources), any pre-planned data assumptions and simplifications	11
Outcomes and prioritization	13	List and define all outcomes for which data will be sought, including prioritization of main and additional outcomes, with rationale	11-12
Risk of bias in individual studies	14	Describe anticipated methods for assessing risk of bias of individual studies, including whether this will be done at the outcome or study level, or both; state how this information will be used in data synthesis	12
Data synthesis	15a	Describe criteria under which study data will be quantitatively synthesised	12
	15b	If data are appropriate for quantitative synthesis, describe planned summary measures, methods of handling data and methods of combining data from studies, including any planned exploration of consistency (such as I^2, Kendall's τ)	NA
	15c	Describe any proposed additional analyses (such as sensitivity or subgroup analyses, meta-regression)	NA
	15d	If quantitative synthesis is not appropriate, describe the type of summary planned	12
Meta-bias(es)	16	Specify any planned assessment of meta-bias(es) (such as publication bias across studies, selective reporting within studies) NA	
Confidence in cumulative evidence	17	Describe how the strength of the body of evidence will be assessed (such as GRADE)	NA

*** It is strongly recommended that this checklist be read in conjunction with the PRISMA-P Explanation and Elaboration (cite when available) for important clarification on the items. Amendments to a review protocol should be tracked and dated.** **The copyright for PRISMA-P (including checklist) is held by the PRISMA-P Group and is distributed under a Creative Commons Attribution Licence 4.0.**

From: Shamseer L, Moher D, Clarke M, Ghersi D, Liberati A, Petticrew M, Shekelle P, Stewart L, PRISMA-P Group. Preferred reporting items for systematic review and meta-analysis protocols (PRISMA-P) 2015: elaboration and explanation. BMJ. 2015 Jan 2;349(jan02 1):g7647.

For peer review only

BMJ Open

Exploring the relationship between local food environments and obesity in UK, Ireland, Australia and New Zealand: A systematic review protocol

Journal:	*BMJ Open*
Manuscript ID	bmjopen-2017-018701.R1
Article Type:	Protocol
Date Submitted by the Author:	10-Nov-2017
Complete List of Authors:	Fuentes Pacheco, Andrea; University of Edinburgh School of Molecular Genetic and Population Health Sciences, Centre of Population Health Sciences, Usher Institute of Population Health Sciences and Informatics<br>Carrillo Balam, Gabriela; University of Edinburgh School of Molecular Genetic and Population Health Sciences, Centre of Population Health Sciences, Usher Institute of Population Health Sciences and Informatics<br>Archibald, Daryll; University of Edinburgh, Scottish Collaboration for Public Health Research & Policy, Usher Institute of Population Health Sciences and Informatics; La Trobe University College of Science Health and Engineering, School of Psychology and Public Health<br>Grant, Elizabeth; The Univesity of Edinburgh, Global Health Academy, Usher Institute of Population Health Sciences and Informatics<br>Skafida, Valeria; University of Edinburgh, Social Policy, School of Social and Political Science
**Primary Subject Heading**:	Public health
Secondary Subject Heading:	Nutrition and metabolism, Epidemiology
Keywords:	obesity, food environments, neighbourhood, food access, systematic review

SCHOLARONE™
Manuscripts

Exploring the relationship between local food environments and obesity in UK, Ireland, Australia and New Zealand: A systematic review protocol

Andrea Fuentes Pacheco[1], Gabriela Carrillo Balam[1], Daryll Archibald[2,3], Liz Grant[4], Valeria Skafida[5]

[1]Centre for Population Health Sciences, Usher Institute of Population Health Sciences and Informatics, The University of Edinburgh, Edinburgh, UK.

[2]Scottish Collaboration for Public Health Research & Policy, Usher Institute of Population Health Sciences and Informatics, The University of Edinburgh, Edinburgh, UK

[3]School of Psychology and Public Health, College of Science, Health and Engineering, La Trobe University, Bundoora, Melbourne

[4]Global Health Academy, Usher Institute of Population Health Sciences and Informatics, The University of Edinburgh, Edinburgh, UK

[5]Social Policy, School of Social and Political Science, The University of Edinburgh, UK.

Corresponding author details

Andrea Fuentes Pacheco

Centre for Population Health Sciences

Usher Institute of Population Health Sciences and Informatics

The University of Edinburgh

Room no. 815, Doorway 3, Old Medical School

For peer review only - http://bmjopen.bmj.com/site/about/guidelines.xhtml

Teviot Place, Edinburgh EH8 9AG

United Kingdom

E-mail: Andrea.Fuentes@ed.ac.uk

Tel: +44(0)131 650 3214

Keywords: obesity, food environments, neighbourhoods, deprivation, food access, food availability, healthy food, unhealthy food, systematic review

Word count: 2510

For peer review only

For peer review only - http://bmjopen.bmj.com/site/about/guidelines.xhtml

ABSTRACT

Introduction: Obesity is a global pandemic that affects all socioeconomic strata, however, the highest figures have been observed in the most disadvantaged social groups. Evidence from the US and Canada showed that specific urban settings encourage obesogenic behaviour in the population living and/or working there. We aim to examine the evidence on the association between local food environments and obesity in the UK, Ireland, Australia and New Zealand.

Methods: Six databases from 1990 to 2017 will be searched: Medline (Ovid), EMBASE (Ovid), Scopus, CINAHL, ASSIA and Web of Science. Grey literature will also be sought by searching Opengrey Europe, The Grey Literature Report and relevant government websites. Additional studies will be retrieved from the reference lists of the selected articles. It will include cohort, longitudinal, case-study and cross-sectional studies that have assessed the relationship between local food environments and obesity in the UK, Ireland, Australia and New Zealand regardless of sex, age and ethnicity of the population. Two researchers will independently select the studies and extract the data. Data items will incorporate: author names, title, study design, year of study, year exposure data collected, country, city, urban/rural, age range, study exclusions, special characteristics of study populations, aims, working definitions of food environments and food outlets, exposure and methods of data collection, outcomes, and key findings. A narrative synthesis and a summary of the results will be produced separately for children and adults, according to the type of food exposure–outcome. All the selected studies will be assessed using The Quality Assessment Tool for Observational Cohort and Cross-Sectional Studies.

For peer review only - http://bmjopen.bmj.com/site/about/guidelines.xhtml

Ethics and dissemination: this study will be based on published literature, and hence there is no need for Ethics approval. Our findings will be presented at relevant national and international scientific conferences and published in a peer-reviewed journal.

Strengths and limitations of this study

- This is the first systematic review to analyse the evidence on the relationship between local food environments and obesity in the UK, Ireland, Australia and New Zealand and to compare the findings with the North American results of a previously conducted review.
- It will incorporate price as a variable of the food environment and an additional analysis of the available foodstuff quality (healthy and unhealthy) inside food outlets.
- We have identified four countries with high obesity figures to conduct the review.

For peer review only

For peer review only - http://bmjopen.bmj.com/site/about/guidelines.xhtml

INTRODUCTION

The obesity pandemic has been increasing dramatically on a global scale since 1980 [1, 2]. According to the World Health Organization (WHO), over 600 million people worldwide were obese in 2014 [2]. The increased trend has particularly affected high and upper-middle-income countries that have reached a high industrialization and urbanization level. However, a growing number of low and middle-income countries are also dealing with this pandemic and the additional burden of malnutrition [3-6]. While the prevalence is rising across all the population segments, the highest figures have been observed in the most disadvantaged social groups [5,6]. Obesity is now a major global health challenge especially among those who suffer socioeconomic disparities across the globe [5,6].

This condition, regarded in some literature as a disease [7] is also described as one of the most complex health problems of modernity due to its multi-causal etiology [7,8]. Beyond individual determinants, it has been proposed that the complex social and physical contexts in which individual behavioural decisions are made strongly influence the outcome [9,10]. Based on this approach, Swinburn and Egger defined the concept of the obesogenic environment as the "sum of the influences that the surroundings, opportunities or conditions of life have on promoting obesity in individuals and populations" [11]**.** Understanding the environmental influences over people's eating behaviours is a major challenge for researchers because people live and function in multiple urban areas where there are many opportunities to shop and eat food throughout the day [12]. Though some studies have found a negative or null association between food environments and obesity [13,14], other studies do point to evidence that deprived food environments encourage an obesogenic food behaviour in the population living and/or working there [8,10,13,15,16]. Studies from a residential perspective have described how these environments appear to encourage an excessive energy intake and

For peer review only - http://bmjopen.bmj.com/site/about/guidelines.xhtml

weight gain in the medium and long term, especially in those residents with less mobility such as the elderly and those on low-incomes, but more work needs to be done to better understand these associations and in particular if there are types of food, for example high-energy-dense foods that are featuring excessively in diets [8,10,13-16].

Local food environments involve social, macro-level and physical aspects that influence people´s food choices [17]. One factor related to the macro-level dimension and two related to the physical aspects have been highlighted as key determinants for those who purchase food within deprived neighbourhoods: price, physical access and availability to (and quality of) foodstuffs inside food sources [17,18]. Food prices, which are mainly regulated by the governments and the global market, can become major barriers for low-income populations [17,18]. Drewnowsky and other authors have identified that individuals under economic constraints, frequently shop and consume high-energy-dense foods which are generally cheaper than healthy products [17,18]. Physical access, which refers to the distance from households to food sources, has also been identified as a barrier for economically and physically disadvantaged people. Studies have described how such people often rely on the purchase of food in nearby and walkable areas rather than spending budgets on public or private transportation to purchase food further away [13,17]. Cummins, Gibson, and Burgoine among other authors, have shown that many deprived urban zones have a major density and exposure of less healthy food sources, increasing the access to high-energy-dense foods [8,13,15,16,19-21]. The in-store availability, depicted as the variety of food provision within food sources, is directly related to the quality of foodstuffs people can purchase. Provision of foods is different in affluent versus deprived areas, where in the latter the offer is frequently less healthy and less varied than in wealthier areas [8, 19-21]. The intersection of these three determinants could facilitate obesogenic food purchases and food intake, leading to a steady increase in body fat over time [8,17-21].

For peer review only - http://bmjopen.bmj.com/site/about/guidelines.xhtml

A large volume of evidence has been generated in North America on this topic during the last decade. According to Cobb et al., in spite of many studies having found a null or negative relationship, a substantial number of other studies have shown a positive association between the aforementioned food environment variables and the prevalence of obesity among children and adults [13,17]. In the case of Europe and Oceania, there is a lack of recent analysis of the emerging evidence despite the UK, Ireland, Australia and New Zealand being among the nations with the highest figures and the worst projections of obesity in Western Europe and Oceania [4]. Two important similarities between these countries and the North American scenario also lead us to believe that a discussion of the studies assessing this relationship is necessary. In all regions the obesity prevalence is concentrated in disadvantaged urban areas, and all of them are experiencing the same post-nutritional transition with a strong influence of a globalized and industrialized food market [6,18]. Therefore this systematic review of the literature in the UK, Ireland, Australia and New Zealand is timely, alongside a comparison with the North American findings.

Eight systematic reviews and one scoping review, focusing mainly on the US and Canada have examined the associations between environment and obesity [10,13,14,22-26]. Two analyzed the whole built environment, including transportation and physical activity access [14, 25]. Three explored the consumer retail food environments without a detailed analysis of the relationship with obesity [10,23,24]. Finally, one examined the association between the food environment and weight status and the other two the relationship with the obesity [13,22,26]. This will be the first systematic review to explore the relationship between local food environments and obesity in UK, Ireland, Australia and New Zealand, incorporating food price [11,18] as part of the food environment. An additional analysis of each variable will be carried out considering the quality of foodstuff (healthy and unhealthy) to which people are exposed in residential zones.

For peer review only - http://bmjopen.bmj.com/site/about/guidelines.xhtml

Cummins and Macintyre (2006) recommend a regional analysis of the available research in other high-income countries in order to identify if the social, economic and geographic contexts are similar enough to attribute the same causes at a global scale [9,10,26]. Until now, the most consistent evidence for a "contextual" effect of food environment is only available from North America [9,13,14,26]. Furthermore, following a variety of public health and private interventions created to promote healthy lifestyle within neighbourhoods over the last decade, such as the insertion of a higher number of supermarkets in deprived areas and social media campaigns about healthy eating [27], it is important to discuss if and how this phenomenon is affecting the most vulnerable population living in these European and Oceanian countries mentioned above.

Objectives

The primary objective is to examine the evidence on the association between local food environments and obesity in the UK, Ireland, Australia and New Zealand. The secondary objectives are to identify gaps in the evidence related to this particular association and analyse the pertinence of this relationship considering the regional contexts.

METHODS

We will carry out a systematic review of the literature. We will draw on the methodology developed by Cobb et al. in 2015 who explored the relationship between local food environments and obesity in the US and Canada [13]. The proposed review will extend the geographic scope of that work by focusing on studies conducted in the UK, Ireland, Australia and New Zealand. It will maintain the physical food access and availability dimensions included in the review by Cobb et al. and will incorporate a third dimension: food price.

For peer review only - http://bmjopen.bmj.com/site/about/guidelines.xhtml

For peer review only

Finally, it will be guided by The Preferred Reporting Items for Systematic Reviews and Meta-Analysis Protocols (PRISMA - P) [28].

Eligibility criteria

Types of studies

All observational epidemiological studies that have assessed access and/or availability of food sources inside neighbourhoods (cohort, longitudinal, case - study and cross-sectional) with group level data and with individual-level data on more than 200 people will be included. It will exclude the studies with less than 200 people. This sample size –threshold was used by Cobb et al. who identified that studies with a smaller sample would be statistically underpowered for the detection of a significant association between the variables [13]. Our study will follow the same criterion as we wish to compare both reviews in a later discussion.

Participants

Eligible participants will include populations regardless of age, sex or ethnicity living in UK, Ireland, Australia and New Zealand. Studies with a separated analysis of adults and children will be included, and in the case where this information has not been provided, the authors will be contacted to request that specific data.

Years considered

The review will include studies from January 1990 through May 2017. The initial cut-point year was adopted by Cobb and other reviews, the rationale being that before the last decade in the 20th century very little data appeared in this field [11, 13, 29].

For peer review only - http://bmjopen.bmj.com/site/about/guidelines.xhtml

Setting

The sample will include research articles only from UK, Ireland, Australia and New Zealand.

Language

Only articles written in English will be included as English is the predominant language of the selected countries.

Exclusion criteria

Literature exclusively looking at individuals with major pathologies, pregnant women, homeless populations, breastfeeding women and participants with physical limitations will be excluded. This is because these conditions independently affect nutritional status. Individuals with obesity grade 3 will also be excluded because this is the most severe stage of obesity and according to the evidence [2,7], there are other physiological causes involved in that status (increases in morbid obesity).

For peer review only

For peer review only - http://bmjopen.bmj.com/site/about/guidelines.xhtml

Search strategy

A preliminary scan in Medline was carried out with the purpose of identifying and building a list of index and free terms (see Supplementary Appendix 1). The final list of search terms was agreed through a consultative process with the review team, clinical and social science colleagues, and a senior librarian at The University of Edinburgh. Due to the iterative nature of the search process, additional search terms and sources may be incorporated into the search strategy. The following databases will be searched: MEDLINE (Ovid), EMBASE (Ovid), Scopus, CINAHL, ASSIA and Web of Science. Grey literature will also be sought by searching Opengrey Europe (SIGLE), The Grey Literature Report, relevant government websites related to the countries included in this review (UK Foresight programme, Australian Institute of Health and Welfare, National library of Australia, PANDORA (Australian Government Web Archive) and Obesity Policy Coalition Australia) and other international organizations' websites related to this topic (World Obesity Federation, Spotlight project and European Association of the Study of Obesity (EASO). Additionally, the reference lists of the selected articles will be checked for additional articles that can potentially be retrieved.

Study records

Data management

Retrieved studies from databases, grey literature and hand – searching will be exported to Endnote Library. The programme will also be used for the screening and deduplication process.

For peer review only - http://bmjopen.bmj.com/site/about/guidelines.xhtml

Selection process

Two researchers (AF and GC) will independently undertake the selection of studies, and data extraction. Discrepancies will be solved by consensus between the two authors. If required, a third party (EG) will make a judgment on the data entered and act as an arbitrator. Full text articles will be retrieved.

Data extraction

Data items were selected by a review of previous data collection strategies published in previous reviews [13, 26] and considering the variables this study seeks to explore through the evidence (See Supplementary Appendix 2). Features to be extracted will include:

- Authors´ names
- Article description
- Design: title, study design, year of study, year exposure data collected, country, city, urban/rural, age range, study exclusions, special characteristics of study population
- Aims and working definitions of food environments and food outlets
- Exposure and methods of data collection
- Outcomes reported: outcome definition, self-reported or measured
- Statistical analysis
- Key findings
- Limitations

Data from each study will be collated into the form, and a final database of all forms will be elaborated using a customised Excel sheet. The extraction form will be piloted before its full

For peer review only - http://bmjopen.bmj.com/site/about/guidelines.xhtml

use in the review. During the pilot, the first ten articles will be independently extracted and jointly reviewed by the two reviewers. The remaining articles will be extracted by one reviewer and reviewed for accuracy by the other.

Outcomes and prioritisation

The primary outcome is obesity. The diagnosis of this pathology follows the criterion established by the World Health Organization to classify the obesity with a Body Mass Index (BMI) over 30 kg/m^2 [2]. Change in BMI, as well as measurements of weight and height will be used to calculate and classify the nutritional status.

The secondary outcome is central obesity, represented by waist circumference and waist to hip ratio (only if the primary outcome is not included).

Risk of bias in individual studies

All the selected studies will be assessed using The Quality Assessment Tool for Observational Cohort and Cross-Sectional Studies [30]. This checklist is a useful tool to develop a critical appraisal of these type of studies. The items contained are:

- Research question
- Study population
- Sample size
- Confounders
- Selection bias
- Data collection methods
- Exposure and outcomes measures
- Blinding of outcome assessors

For peer review only - http://bmjopen.bmj.com/site/about/guidelines.xhtml

- Statistical analyses

Data synthesis

A separate narrative synthesis and a summary of the results for children and adults will be performed, according to the type of food exposure-outcome. The measurement techniques chosen in every study will also be analysed. Finally, this will be compared with the main findings generated in the original North American systematic review.

Subgroups analysis: men, women, adults, children, low SES, high SES, urban, rural and by country.

Protocol registration

A detailed protocol was registered with the International Prospective Register of Systematic Reviews (PROSPERO): 2017:CRD42017068193 (http://www.crd.york.ac.uk/PROSPERO/display_record.asp?ID=CRD42017068193). The study will be guided by the Preferred Reporting Items for Systematic review and Meta-Analysis Protocols (PRISMA-P) 2015 statement [28].

CONCLUSIONS

Previous systematic reviews have assessed the evidence related to the association between local food environments and obesity in the US and Canada [13,26]. This systematic review is the first study that will geographically extend the work undertaken by Cobb et al., analysing the relationship on evidence from the UK, Ireland, Australia and New Zealand. Furthermore, it will incorporate food price as part of the food environment and an additional analysis of the quality of foodstuff (healthy and unhealthy) available inside the residential areas measured.

For peer review only - http://bmjopen.bmj.com/site/about/guidelines.xhtml

For peer review only

Finally, it will undertake a comparative analysis by country and between the regional results and the North American findings. This is highly relevant in order to gain a better understanding of whether the phenomenon is subject to regional variations or if it is occurring on a global scale.

ETHICS AND DISSEMINATION

As this is a review of published literature ethics approval has not been sought. However, this work is subject to Institutional Review Board oversight by The University of Edinburgh's Centre for Population Health Sciences. The review findings will be presented in relevant national and international scientific conferences and be published in a peer-reviewed journal.

For peer review only

Competing interests

None declared.

Funding

DA is supported jointly by the Medical Research Council (MRC; MR/K023209/1) and the Chief Scientist Office (CSO).

Contributors

Andrea Fuentes conceived the idea for this work and drafted the protocol under the supervision of Daryll Archibald, Liz Grant and Valeria Skafida. Gabriela Carrillo suggested valuable corrections to the manuscript. All the authors will be involved in the systematic review process.

For peer review only - http://bmjopen.bmj.com/site/about/guidelines.xhtml

REFERENCES

1. Mendis S, Pekka P, Norrving, B, et al. Global Atlas on cardiovascular disease, prevention and control. World Health Organization in collaboration with the World Heart Federation and the World Stroke Organization, c2011.

2. World Health Organization. Fact sheets: Obesity and overweight. [Cited 4 October 2017]. http://www.who.int/mediacentre/factsheets/fs311/en/.

3. Popkin BM. The Nutrition Transition: An Overview of World Patterns of Change. Nutr Rev. 2004;62(7):S140-S3.

4. *OECD (2010).* Obesity and the Economics of Prevention: Fit not Fat*, OECD Publishing, Paris.*

5. Popkin BM, Adair L, Wen Ng S. NOW AND THEN: The Global Nutrition Transition: The Pandemic of Obesity in Developing Countries. Nutr Rev. 2012;70(1):3–21.

6. NCD Risk Factor Collaboration (NCD-RisC)*. Worldwide trends in body-mass index, underweight, overweight, and obesity from 1975 to 2016: a pooled analysis of 2416 population-based measurement studies in 128·9 million children, adolescents, and adults. Lancet. 2017. DOI: http://dx.doi.org/10.1016/S0140-6736(17)32129-3.

7. Jensen M, Ryan D, Apovian C, et al. 2013 AHA/ACC/TOS Guideline for the Management of Overweight and Obesity in Adults: A Report of the American College of Cardiology/American Heart Association Task Force on Practice Guidelines and The Obesity Society. J Am Coll Cardiol. 2014 Jul 1;63(25 Pt B):2985-3023.

8. Short Science Reviews. Foresight Tackling Obesities: Future Choices. Obesity Reviews. 8(s1)v–210.

9. Cummins S, Mcintyre S. Food environments and obesity - neighbourhood or nation? Int J Epidemiol. 2006;35:100-4.

For peer review only - http://bmjopen.bmj.com/site/about/guidelines.xhtml

10. Giskes K, Van Lenthe M, Avendano – Pabon M, Brug J. A systematic review of environmental factors and obesogenic dietary intakes among adults: are we getting closer to understanding obesogenic environments?. Obesity reviews (2011) 12, e95-e106.

11. Swinburn B, Egger G, Raza F. Dissecting Obesogenic Environments: The Development and Application of a Framework for Identifying and Prioritizing Environmental Interventions for Obesity. Prev Med 1999;29((6 Pt 1)):563-70.

12. Rainham D, McDowell I, Krewski D, Sawada M. Conceptualizing the Healthscape: contributions of Time Geography, location, technologies and spatial ecology to place and health research. Soc. Sci. Med 2010, 70, 668-676.

13. Cobb L, Appel L, Franco M, et al. The relationship of the local food environment with obesity: A systematic review of methods, study quality and results. Obesity (Silver Spring). 2015;23(7):1331–44.

14. Feng J, Glass T, Curriero F, et al. The built environment and obesity: A systematic review of the epidemiologic evidence. Health & Place 16 (2010) 175 – 190.

15. Lake A, Townshend T. Obesogenic environments: exploring the built and food environments. J R Soc Promot Health. 2006;126(6):262-7.

16. Short Science Reviews. Foresight Tackling Obesities: Future Choices – Obesogenic Environments – Evidence Review. Government Office for Science, 2007.

17. Story M, Kaphingst K, Robinson – O'brien R, Glanz K. Creating healthy food and eating environments: policy and environmental approaches. Annu. Rev. Public Health 2008. 29:253–72.

18. Drewnowski A. Obesity, diets, and social inequalities. Nutr Rev. 2009;67(Suppl. 1):S36–S9.

19. Cummins S, McKay L, McIntyre S. McDonald's Restaurants and Neighborhood Deprivation in Scotland and England. Am J Prev Med 2005;29(4):308–310.

For peer review only

For peer review only - http://bmjopen.bmj.com/site/about/guidelines.xhtml

20. Gibson D. The neighborhood food environment and adult weight status: estimates from longitudinal data. Am J Public Health. 2011 Jan;101(1):71-8.

21. Burgoine T, Forouhi N, Griffin S, et al. Associations between exposure to takeaway food outlets, takeaway food consumption, and body weight in Cambridgeshire, UK: population based, cross sectional study. BMJ 2014;348:g1464.

22. Mackenbach JD, Rutter H, Compernolle S, et al. Obesogenic environments: a systematic review of the association between the physical environment and adult weight status, the SPOTLIGHT project. BMC Public Health. 2014;14:233.

23. Minaker LM, Shuh A, Olstad DL, et al. Retail food environments research in Canada: A scoping review. Can J Public Health. 2016;107(0):5344.

24. Ni Mhurchu C, Vandevijvere S, Waterlander W, et al. Monitoring the availability of healthy and unhealthy foods and non-alcoholic beverages in community and consumer retail food environments globally. Obes rev. 2013;14(1):108–19.

25. Papas M, Alberg A, Ewing R, et al. The built environment and obesity. Epidemiol Rev. 2007;29:129-43.

26. Holsten J. Obesity and the community food environment: a systematic review. Public Health Nutr. 2009 Mar;12(3):397-405.

27. Scottish Public Health Network. Obesity Route Map. 1.0 ed: Scottish Government; 2011.

28. Shamseer L, Moher D, Clarke M, et al. PRISMA-P Group. Preferred reporting items for systematic review and meta-analysis protocols (PRISMA-P) 2015: elaboration and explanation. BMJ. 2015;2(349):g7647.

29. Glanz K. Measuring food environments: a historical perspective. Am J Prev Med 2009; 36 (4S).

For peer review only

For peer review only - http://bmjopen.bmj.com/site/about/guidelines.xhtml

30. National Heart, Lung and Blood Institute. Qualitiy Assessment Tool for Observational Cohort and Cross-sectional studies. Systematic Evidence Review from the Blood Pressure Expert Panel, 2011. [Cited 12 October 2017]. https://www.nhlbi.nih.gov/health-pro/guidelines/in-develop/cardiovascular-risk-reduction/tools/cohort.

For peer review only

For peer review only - http://bmjopen.bmj.com/site/about/guidelines.xhtml

Supplementary APPENDIX

Search strategy developed for MEDLINE (OVID)

1. Food environment*.tw
2. Fodscape*.tw
3. Food access.tw
4. Food availability.tw
5. Food affordability.tw
6. Food outlet access.tw
7. Food price.tw
8. Food store*.tw
9. Food outlet*.tw
10. Grocer*.tw
11. Convenience store*.tw
12. Supermarket*.tw
13. Restaurant*.tw
14. Food bank*.tw
15. Online shopping.tw
16. Food shopping.tw
17. Economic access.tw
18. Residential.tw
19. Neighbo*.tw
20. Neighbourhood.tw
21. Urban area*.tw
22. Obes*.tw
23. Obesogenic.tw
24. Overnutrition/
25. Overnutrition.tw
26. Over nutrition.tw
27. Overweight/
28. Body Mass Index/
29. BMI.tw
30. Weight adj2 change.tw.
31. Weight status.tw

For peer review only

32. Weight control.tw
33. Waist circumference.tw
34. OR/1-21
35. OR/22-33
36. 34 AND 35

For peer review only

Preliminary data extraction form

Reviewer identification

Date:

Reference ID:

Name:

Article description

Title:

Authors:

Institution:

Corresponding email:

Type of source (journal article/ grey literature):

Year:

Aims and study design

Aims/objectives:

Study design:

Methodology:

1. Settings

a) Country/city:

b) Urban/rural area:

2. Special characteristics of the population:

a) Age structure:

b) Sex structure:

c) Ethnic features:

d) Educational level:

e) Socioeconomic level:

f) Other characteristics:

For peer review only

3. Study exclusions:<br><br>4. Exposure:<br><br>a) Food environment definition:<br><br>b) Food sources/outlet definition:<br><br>c) Data source<br><br>d) Year exposure data collected:<br><br>e) Type of food environment variable:<br><br>f) Food environment variable definition:<br><br>g) Methods of data collection<br><br>5. Outcomes:<br><br>a) Reported outcomes:<br><br>b) Outcomes definition:<br><br>c) Type of measure (self-reported/measured):
**<u>Statistical Analysis</u>**<br><br>a) Modelling strategy<br>b) Reported bias<br>c) Reported confounders
**<u>Key findings:</u>**<br><br>Main findings:
**<u>Limitations:</u>**<br><br>Study limitations:

For peer review only

PRISMA-P (Preferred Reporting Items for Systematic review and Meta-Analysis Protocols) 2015 checklist: recommended items to address in a systematic review protocol*

Section and topic	Item No	Checklist item	
**ADMINISTRATIVE INFORMATION**			
Title:			
Identification	1a	Identify the report as a protocol of a systematic review	1
Update	1b	If the protocol is for an update of a previous systematic review, identify as such	NA
Registration	2	If registered, provide the name of the registry (such as PROSPERO) and registration number	14
Authors:			
Contact	3a	Provide name, institutional affiliation, e-mail address of all protocol authors; provide physical mailing address of corresponding author	1
Contributions	3b	Describe contributions of protocol authors and identify the guarantor of the review	15
Amendments	4	If the protocol represents an amendment of a previously completed or published protocol, identify as such and list changes; otherwise, state plan for documenting important protocol amendments	NA
Support:			
Sources	5a	Indicate sources of financial or other support for the review	15
Sponsor	5b	Provide name for the review funder and/or sponsor	15
Role of sponsor or funder	5c	Describe roles of funder(s), sponsor(s), and/or institution(s), if any, in developing the protocol	NA
**INTRODUCTION**			
Rationale	6	Describe the rationale for the review in the context of what is already known	5-8
Objectives	7	Provide an explicit statement of the question(s) the review will address with reference to participants, interventions, comparators, and outcomes (PICO)	8
**METHODS**			
Eligibility criteria	8	Specify the study characteristics (such as PICO, study design, setting, time frame) and report characteristics (such as years considered, language, publication status) to be used as criteria for eligibility for the review	9-10
Information sources	9	Describe all intended information sources (such as electronic databases, contact with study authors, trial registers or other grey literature sources) with planned dates of coverage	11
Search strategy	10	Present draft of search strategy to be used for at least one electronic database, including planned limits, such that it could be repeated	11
Study records:			
Data management	11a	Describe the mechanism(s) that will be used to manage records and data throughout the review	11

For peer review only

Selection process	11b	State the process that will be used for selecting studies (such as two independent reviewers) through each phase of the review (that is, screening, eligibility and inclusion in meta-analysis)	11-12
Data collection process	11c	Describe planned method of extracting data from reports (such as piloting forms, done independently, in duplicate), any processes for obtaining and confirming data from investigators	12
Data items	12	List and define all variables for which data will be sought (such as PICO items, funding sources), any pre-planned data assumptions and simplifications	12
Outcomes and prioritization	13	List and define all outcomes for which data will be sought, including prioritization of main and additional outcomes, with rationale	13
Risk of bias in individual studies	14	Describe anticipated methods for assessing risk of bias of individual studies, including whether this will be done at the outcome or study level, or both; state how this information will be used in data synthesis	13
Data synthesis	15a	Describe criteria under which study data will be quantitatively synthesised	14
	15b	If data are appropriate for quantitative synthesis, describe planned summary measures, methods of handling data and methods of combining data from studies, including any planned exploration of consistency (such as I^2, Kendall's τ)	NA
	15c	Describe any proposed additional analyses (such as sensitivity or subgroup analyses, meta-regression)	NA
	15d	If quantitative synthesis is not appropriate, describe the type of summary planned	14
Meta-bias(es)	16	Specify any planned assessment of meta-bias(es) (such as publication bias across studies, selective reporting within studies) NA	
Confidence in cumulative evidence	17	Describe how the strength of the body of evidence will be assessed (such as GRADE)	NA

*** It is strongly recommended that this checklist be read in conjunction with the PRISMA-P Explanation and Elaboration (cite when available) for important clarification on the items. Amendments to a review protocol should be tracked and dated. The copyright for PRISMA-P (including checklist) is held by the PRISMA-P Group and is distributed under a Creative Commons Attribution Licence 4.0.**

From: Shamseer L, Moher D, Clarke M, Ghersi D, Liberati A, Petticrew M, Shekelle P, Stewart L, PRISMA-P Group. Preferred reporting items for systematic review and meta-analysis protocols (PRISMA-P) 2015: elaboration and explanation. BMJ. 2015 Jan 2;349(jan02 1):g7647.

For peer review only

BMJ Open

Exploring the relationship between local food environments and obesity in UK, Ireland, Australia and New Zealand: A systematic review protocol

Journal:	*BMJ Open*
Manuscript ID	bmjopen-2017-018701.R2
Article Type:	Protocol
Date Submitted by the Author:	10-Jan-2018
Complete List of Authors:	Fuentes Pacheco, Andrea; University of Edinburgh School of Molecular Genetic and Population Health Sciences, Centre of Population Health Sciences, Usher Institute of Population Health Sciences and Informatics<br>Carrillo Balam, Gabriela; University of Edinburgh School of Molecular Genetic and Population Health Sciences, Centre of Population Health Sciences, Usher Institute of Population Health Sciences and Informatics<br>Archibald, Daryll; University of Edinburgh, Scottish Collaboration for Public Health Research & Policy, Usher Institute of Population Health Sciences and Informatics; La Trobe University College of Science Health and Engineering, School of Psychology and Public Health<br>Grant, Elizabeth; The Univesity of Edinburgh, Global Health Academy, Usher Institute of Population Health Sciences and Informatics<br>Skafida, Valeria; University of Edinburgh, Social Policy, School of Social and Political Science
**Primary Subject Heading**:	Public health
Secondary Subject Heading:	Nutrition and metabolism, Epidemiology
Keywords:	obesity, food environments, neighbourhood, food access, systematic review

SCHOLARONE™
Manuscripts

Exploring the relationship between local food environments and obesity in UK, Ireland, Australia and New Zealand: A systematic review protocol

Andrea Fuentes Pacheco[1], Gabriela Carrillo Balam[1], Daryll Archibald[2,3], Liz Grant[4], Valeria Skafida[5]

[1]Centre for Population Health Sciences, Usher Institute of Population Health Sciences and Informatics, The University of Edinburgh, Edinburgh, UK.

[2]Scottish Collaboration for Public Health Research & Policy, Usher Institute of Population Health Sciences and Informatics, The University of Edinburgh, Edinburgh, UK

[3]School of Psychology and Public Health, College of Science, Health and Engineering, La Trobe University, Bundoora, Melbourne

[4]Global Health Academy, Usher Institute of Population Health Sciences and Informatics, The University of Edinburgh, Edinburgh, UK

[5]Social Policy, School of Social and Political Science, The University of Edinburgh, UK.

Corresponding author details

Andrea Fuentes Pacheco

Centre for Population Health Sciences

Usher Institute of Population Health Sciences and Informatics

The University of Edinburgh

Room no. 815, Doorway 3, Old Medical School

For peer review only

Teviot Place, Edinburgh EH8 9AG

United Kingdom

E-mail: Andrea.Fuentes@ed.ac.uk

Tel: +44(0)131 650 3214

Keywords: obesity, food environments, neighbourhoods, deprivation, food access, food availability, healthy food, unhealthy food, systematic review

Word count: 2510

For peer review only

For peer review only - http://bmjopen.bmj.com/site/about/guidelines.xhtml

ABSTRACT

Introduction: Obesity is a global pandemic that affects all socioeconomic strata, however, the highest figures have been observed in the most disadvantaged social groups. Evidence from the US and Canada showed that specific urban settings encourage obesogenic behaviour in the population living and/or working there. We aim to examine the evidence on the association between local food environments and obesity in the UK, Ireland, Australia and New Zealand.

Methods: Six databases from 1990 to 2017 will be searched: Medline (Ovid), EMBASE (Ovid), Scopus, CINAHL, ASSIA and Web of Science. Grey literature will also be sought by searching Opengrey Europe, The Grey Literature Report and relevant government websites. Additional studies will be retrieved from the reference lists of the selected articles. It will include cohort, longitudinal, case-study and cross-sectional studies that have assessed the relationship between local food environments and obesity in the UK, Ireland, Australia and New Zealand regardless of sex, age and ethnicity of the population. Two researchers will independently select the studies and extract the data. Data items will incorporate: author names, title, study design, year of study, year exposure data collected, country, city, urban/rural, age range, study exclusions, special characteristics of study populations, aims, working definitions of food environments and food outlets, exposure and methods of data collection, outcomes, and key findings. A narrative synthesis and a summary of the results will be produced separately for children and adults, according to the type of food exposure–outcome. All the selected studies will be assessed using The Quality Assessment Tool for Observational Cohort and Cross-Sectional Studies.

For peer review only - http://bmjopen.bmj.com/site/about/guidelines.xhtml

Ethics and dissemination: this study will be based on published literature, and therefore ethical approval has not been sought. Our findings will be presented at relevant national and international scientific conferences and published in a peer-reviewed journal.

Strengths and limitations of this study

- This is the first systematic review to analyse the evidence on the relationship between local food environments and obesity in the UK, Ireland, Australia and New Zealand and to compare the findings with the North American results of a previously conducted review.
- It will incorporate price as a variable of the food environment. An additional analysis of the available foodstuff quality (healthy and unhealthy) inside food outlets will be completed.
- The review will be conducted in four countries with high obesity figures.
- As we cannot cover the whole European and Oceanic regions we selected four countries to represent both continents.

For peer review only

INTRODUCTION

The obesity pandemic has been increasing dramatically on a global scale since 1980 [1, 2]. According to the World Health Organization (WHO), over 600 million people worldwide were obese in 2014 [2]. The increased trend has particularly affected high and upper-middle-income countries that have reached a high industrialization and urbanization level. However, a growing number of low and middle-income countries are also dealing with this pandemic alongside the additional burden of malnutrition [3-6]. While the prevalence is rising across all the population segments, the highest figures have been observed in the most disadvantaged social groups [5,6]. Obesity is now a major global health challenge especially among those who suffer socioeconomic disparities across the globe [5,6].

This condition, regarded in some literature as a disease [7] has been described as one of the most complex health problems of modernity due to its multi-causal etiology [7,8]. Beyond individual determinants, the complex social and physical contexts in which individual behavioural decisions are made appear to strongly influence the outcome [9,10]. Based on this approach, Swinburn and Egger defined the concept of the obesogenic environment as the "sum of the influences that the surroundings, opportunities or conditions of life have on promoting obesity in individuals and populations" [11]**.** Understanding the environmental influences over people's eating behaviours is a major challenge for researchers because people live and function in multiple urban areas where there are many opportunities to shop and eat food throughout the day [12]. Though some studies have found a negative or null association between food environments and obesity [13,14], other studies do point to evidence that deprived food environments encourage an obesogenic food behaviour in the population living and/or working there [8,10,13,15,16]. Studies from a residential perspective

For peer review only - http://bmjopen.bmj.com/site/about/guidelines.xhtml

have described how these environments appear to encourage an excessive energy intake and weight gain in the medium and long term, especially in those residents whose mobility is limited because of health or transport access such as the elderly and those on low-incomes, but more work needs to be done to better understand these associations and in particular if there are types of food, for example high-energy-dense foods that are featuring excessively in diets [8,10,13-16].

Local food environments include the social, macro-level and physical aspects that influence people´s food choices [17]. One factor related to the macro-level dimension and two related to the physical aspects have been highlighted as key determinants for those who purchase food within deprived neighbourhoods: price, physical access and availability to (and quality of) foodstuffs inside food sources [17,18]. Food prices, which are mainly regulated by the governments and the global market, can become major barriers for low-income populations [17,18]. Drewnowsky and other authors have identified that individuals under economic constraints, frequently shop and consume high-energy-dense foods which are generally cheaper than healthy products [17,18]. Physical access, which refers to the distance from households to food sources, has also been identified as a barrier for economically and physically disadvantaged people. Studies have described how such people often rely on the purchase of food in nearby and walkable areas rather than spending budgets on public or private transportation to purchase food further away [13,17]. Cummins, Gibson, and Burgoine among others, have shown that many deprived urban zones have a major density and exposure of less healthy food sources, increasing the access to high-energy-dense foods [8,13,15,16,19-21]. The in-store availability, depicted as the variety of food provision within food sources, is directly related to the quality of foodstuffs people can purchase. Provision of foods is different in affluent versus deprived areas, where in the latter the offer is frequently less healthy and less varied than in wealthier areas [8, 19-21]. The intersection of these three

For peer review only - http://bmjopen.bmj.com/site/about/guidelines.xhtml

determinants could facilitate obesogenic food purchases and food intake, leading to a steady increase in body fat over time [8,17-21].

A large volume of evidence has been generated in North America on this topic during the last decade. According to Cobb et al., in spite of many studies having found a null or negative relationship, a substantial number of other studies have shown a positive association between the aforementioned food environment variables and the prevalence of obesity among children and adults [13,17]. In the case of Europe and Oceania, there is a lack of recent analysis of the emerging evidence despite the UK, Ireland, Australia and New Zealand being among the nations with the highest figures and the worst projections of obesity in Western Europe and Oceania [4]. Two important similarities between these countries and the North American scenario also lead us to believe that a discussion of the studies assessing this relationship is necessary. In all regions the obesity prevalence is concentrated in disadvantaged urban areas, and all of them are experiencing the same post-nutritional transition with a strong influence of a globalized and industrialized food market [6,18]. Therefore this systematic review of the literature in the UK, Ireland, Australia and New Zealand is timely, alongside a comparison with the North American findings.

Eight systematic reviews and one scoping review, focusing mainly on the US and Canada have examined the associations between environment and obesity [10,13,14,22-26]. Two analyzed the whole built environment, including transportation and physical activity access [14, 25]. Three explored the consumer retail food environments without a detailed analysis of the relationship with obesity [10,23,24]. Finally, one examined the association between the food environment and weight status and the other two the relationship with the obesity [13,22, 26]. This will be the first systematic review to explore the relationship between local food environments and obesity in UK, Ireland, Australia and New Zealand, incorporating food price [11,18] as part of the food environment. An additional analysis of each variable

For peer review only - http://bmjopen.bmj.com/site/about/guidelines.xhtml

For peer review only

will be carried out considering the quality of foodstuff (healthy and unhealthy) to which people are exposed in residential zones.

Cummins and Macintyre (2006) recommend a regional analysis of the available research in other high-income countries in order to identify if the social, economic and geographic contexts are similar enough to attribute the same causes at a global scale [9,10,26]. Until now, the most consistent evidence for a "contextual" effect of food environment is only available from North America [9,13,14,26]. Furthermore, following a variety of public health and private interventions created to promote healthy lifestyle within neighbourhoods over the last decade, such as the insertion of a higher number of supermarkets in deprived areas and social media campaigns about healthy eating [27], it is important to discuss if and how this phenomenon is affecting the most vulnerable population living in these European and Oceanian countries mentioned above.

Objectives

The primary objective is to examine the evidence on the association between local food environments and obesity in the UK, Ireland, Australia and New Zealand. The secondary objectives are to identify gaps in the evidence related to this particular association and analyse the pertinence of this relationship considering the regional contexts.

METHODS

We will carry out a systematic review of the literature. We will draw on the methodology developed by Cobb et al. in 2015 who explored the relationship between local food environments and obesity in the US and Canada [13]. The proposed review will extend the geographic scope of that work by focusing on studies conducted in the UK, Ireland, Australia

For peer review only - http://bmjopen.bmj.com/site/about/guidelines.xhtml

and New Zealand. It will maintain the physical food access and availability dimensions included in the review by Cobb et al. and will incorporate a third dimension: food price. Finally, it will be guided by The Preferred Reporting Items for Systematic Reviews and Meta-Analysis Protocols (PRISMA - P) [28].

Eligibility criteria

Types of studies

All observational epidemiological studies that have assessed access and/or availability of food sources inside neighbourhoods (cohort, longitudinal, case - study and cross-sectional) with group level data and with individual-level data on more than 200 people will be included. It will exclude the studies with less than 200 people. This sample size –threshold was used by Cobb et al. who identified that studies with a smaller sample would be statistically underpowered for the detection of a significant association between the variables [13]. Our study will follow the same criterion as we wish to compare both reviews in a later discussion.

Participants

Eligible participants will include populations regardless of age, sex or ethnicity living in UK, Ireland, Australia and New Zealand. Studies with a separated analysis of adults and children will be included, and in the case where this information has not been provided, the authors will be contacted to request that specific data.

For peer review only - http://bmjopen.bmj.com/site/about/guidelines.xhtml

Years considered

The review will include studies from January 1990 through May 2017. The initial cut-point year was adopted by Cobb and other reviews, the rationale being that before the last decade of the 20th century very little data appeared in this field [11, 13, 29].

Setting

The sample will include research articles only from UK, Ireland, Australia and New Zealand.

Language

Only articles written in English will be included as English is the predominant language of the selected countries.

Exclusion criteria

Literature exclusively looking at individuals with major pathologies, pregnant women, homeless populations, breastfeeding women and participants with physical limitations will be excluded. This is because these conditions independently affect nutritional status. Individuals with obesity grade 3 will also be excluded because this is the most severe stage of obesity and according to the evidence [2,7], there are other physiological causes involved in that status (increases in morbid obesity).

For peer review only - http://bmjopen.bmj.com/site/about/guidelines.xhtml

Search strategy

A preliminary scan in Medline was carried out with the purpose of identifying and building a list of index and free terms (see Supplementary Appendix 1). The final list of search terms was agreed through a consultative process with the review team, clinical and social science colleagues, and a senior librarian at The University of Edinburgh. Due to the iterative nature of the search process, additional search terms and sources may be incorporated into the search strategy. The following databases will be searched: MEDLINE (Ovid), EMBASE (Ovid), Scopus, CINAHL, ASSIA and Web of Science. Grey literature will also be sought by searching Opengrey Europe (SIGLE), The Grey Literature Report, relevant government websites related to the countries included in this review (UK Foresight programme, Australian Institute of Health and Welfare, National library of Australia, PANDORA (Australian Government Web Archive) and Obesity Policy Coalition Australia) and other international organizations' websites related to this topic (World Obesity Federation, Spotlight project and European Association of the Study of Obesity (EASO). Additionally, the reference lists of the selected articles will be checked for additional articles that can potentially be retrieved.

Study records

Data management

Retrieved studies from databases, grey literature and hand – searching will be exported to Endnote Library. The programme will also be used for the screening and deduplication process.

For peer review only - http://bmjopen.bmj.com/site/about/guidelines.xhtml

Selection process

Two researchers (AF and GC) will independently undertake the selection of studies, and data extraction. Discrepancies will be solved by consensus between the two authors. If required, a third party (EG) will make a judgment on the data entered and act as an arbitrator. Full text articles will be retrieved.

Data extraction

Data items were selected after a review of previous data collection strategies published in previous reviews [13, 26] and through consideration of the variables this study seeks to explore through the evidence (See Supplementary Appendix 2). Features to be extracted will include:

- Authors´ names
- Article description
- Design: title, study design, year of study, year exposure data collected, country, city, urban/rural, age range, study exclusions, special characteristics of study population
- Aims and working definitions of food environments and food outlets
- Exposure and methods of data collection
- Outcomes reported: outcome definition, self-reported or measured
- Statistical analysis
- Key findings
- Limitations

For peer review only - http://bmjopen.bmj.com/site/about/guidelines.xhtml

Data from each study will be collated into the form, and a final database of all forms will be elaborated using a customised Excel sheet. The extraction form will be piloted before its full use in the review. During the pilot, the first ten articles will be independently extracted and jointly reviewed by the two reviewers. The remaining articles will be extracted by one reviewer and reviewed for accuracy by the other.

Outcomes and prioritisation

The primary outcome is obesity. The diagnosis of this pathology follows the criterion established by the World Health Organisation to classify the obesity with a Body Mass Index (BMI) over 30 kg/m² [2]. Change in BMI, as well as measurements of weight and height will be used to calculate and classify the nutritional status.

The secondary outcome is central obesity, represented by waist circumference and waist to hip ratio (only if the primary outcome is not included).

Risk of bias in individual studies

All the selected studies will be assessed using The Quality Assessment Tool for Observational Cohort and Cross-Sectional Studies [30]. This checklist is a useful tool to develop a critical appraisal of these type of studies. The items contained are:

- Research question
- Study population
- Sample size
- Confounders
- Selection bias
- Data collection methods

For peer review only - http://bmjopen.bmj.com/site/about/guidelines.xhtml

- Exposure and outcomes measures
- Blinding of outcome assessors
- Statistical analyses

Data synthesis

A separate narrative synthesis and a summary of the results for children and adults will be performed, according to the type of food exposure-outcome. The measurement techniques chosen in every study will also be analysed. Finally, this will be compared with the main findings generated in the original North American systematic review.

Subgroups analysis: men, women, adults, children, low SES, high SES, urban, rural and by country.

Protocol registration

A detailed protocol was registered with the International Prospective Register of Systematic Reviews (PROSPERO): 2017:CRD42017068193 (http://www.crd.york.ac.uk/PROSPERO/display_record.asp?ID=CRD42017068193). The study will be guided by the Preferred Reporting Items for Systematic review and Meta-Analysis Protocols (PRISMA-P) 2015 statement [28].

CONCLUSIONS

Previous systematic reviews have assessed the evidence related to the association between local food environments and obesity in the US and Canada [13,26]. This systematic review is the first study that will geographically extend the work undertaken by Cobb et al., analysing the relationship on evidence from the UK, Ireland, Australia and New Zealand. Furthermore,

For peer review only - http://bmjopen.bmj.com/site/about/guidelines.xhtml

it will incorporate food price as part of the food environment and develop an additional analysis of the quality of foodstuff (healthy and unhealthy) available inside the residential areas measured. Finally, it will undertake a comparative analysis by country and between the regional results and the North American findings. This is highly relevant in order to gain a better understanding of whether the phenomenon is subject to regional variations or if it is occurring on a global scale.

ETHICS AND DISSEMINATION

As this is a review of published literature ethics approval has not been sought. However, this work is subject to Institutional Review Board oversight by The University of Edinburgh's Centre for Population Health Sciences. The review findings will be presented in relevant national and international scientific conferences and be published in a peer-reviewed journal.

Competing interests

None declared.

Funding

DA is supported jointly by the Medical Research Council (MRC; MR/K023209/1) and the Chief Scientist Office (CSO).

Contributors

Andrea Fuentes conceived the idea for this work and drafted the protocol. The draft was critically revised according to several rounds of critical comments by Daryll Archibald, Liz Grant and Valeria Skafida and Gabriela Carrillo. All the authors will be involved in the systematic review process.

For peer review only - http://bmjopen.bmj.com/site/about/guidelines.xhtml

REFERENCES

1. Mendis S, Pekka P, Norrving, B, et al. Global Atlas on cardiovascular disease, prevention and control. World Health Organization in collaboration with the World Heart Federation and the World Stroke Organization, c2011.

2. World Health Organization. Fact sheets: Obesity and overweight. [Cited 4 October 2017]. http://www.who.int/mediacentre/factsheets/fs311/en/.

3. Popkin BM. The Nutrition Transition: An Overview of World Patterns of Change. Nutr Rev. 2004;62(7):S140-S3.

4. *OECD (2010).* Obesity and the Economics of Prevention: Fit not Fat*, OECD Publishing, Paris.*

5. Popkin BM, Adair L, Wen Ng S. NOW AND THEN: The Global Nutrition Transition: The Pandemic of Obesity in Developing Countries. Nutr Rev. 2012;70(1):3–21.

6. NCD Risk Factor Collaboration (NCD-RisC)*. Worldwide trends in body-mass index, underweight, overweight, and obesity from 1975 to 2016: a pooled analysis of 2416 population-based measurement studies in 128·9 million children, adolescents, and adults. Lancet. 2017. DOI: http://dx.doi.org/10.1016/S0140-6736(17)32129-3.

7. Jensen M, Ryan D, Apovian C, et al. 2013 AHA/ACC/TOS Guideline for the Management of Overweight and Obesity in Adults: A Report of the American College of Cardiology/American Heart Association Task Force on Practice Guidelines and The Obesity Society. J Am Coll Cardiol. 2014 Jul 1;63(25 Pt B):2985-3023.

8. Short Science Reviews. Foresight Tackling Obesities: Future Choices. Obesity Reviews. 8(s1)v–210.

For peer review only - http://bmjopen.bmj.com/site/about/guidelines.xhtml

9. Cummins S, Mcintyre S. Food environments and obesity - neighbourhood or nation? Int J Epidemiol. 2006;35:100-4.

10. Giskes K, Van Lenthe M, Avendano – Pabon M, Brug J. A systematic review of environmental factors and obesogenic dietary intakes among adults: are we getting closer to understanding obesogenic environments?. Obesity reviews (2011) 12, e95-e106.

11. Swinburn B, Egger G, Raza F. Dissecting Obesogenic Environments: The Development and Application of a Framework for Identifying and Prioritizing Environmental Interventions for Obesity. Prev Med 1999;29((6 Pt 1)):563-70.

12. Rainham D, McDowell I, Krewski D, Sawada M. Conceptualizing the Healthscape: contributions of Time Geography, location, technologies and spatial ecology to place and health research. Soc. Sci. Med 2010, 70, 668-676.

13. Cobb L, Appel L, Franco M, et al. The relationship of the local food environment with obesity: A systematic review of methods, study quality and results. Obesity (Silver Spring). 2015;23(7):1331–44.

14. Feng J, Glass T, Curriero F, et al. The built environment and obesity: A systematic review of the epidemiologic evidence. Health & Place 16 (2010) 175 – 190.

15. Lake A, Townshend T. Obesogenic environments: exploring the built and food environments. J R Soc Promot Health. 2006;126(6):262-7.

16. Short Science Reviews. Foresight Tackling Obesities: Future Choices – Obesogenic Environments – Evidence Review. Government Office for Science, 2007.

17. Story M, Kaphingst K, Robinson – O'brien R, Glanz K. Creating healthy food and eating environments: policy and environmental approaches. Annu. Rev. Public Health 2008. 29:253–72.

18. Drewnowski A. Obesity, diets, and social inequalities. Nutr Rev. 2009;67(Suppl. 1):S36–S9.

For peer review only - http://bmjopen.bmj.com/site/about/guidelines.xhtml

19. Cummins S, McKay L, McIntyre S. McDonald's Restaurants and Neighborhood Deprivation in Scotland and England. Am J Prev Med 2005;29(4):308–310.

20. Gibson D. The neighborhood food environment and adult weight status: estimates from longitudinal data. Am J Public Health. 2011 Jan;101(1):71-8.

21. Burgoine T, Forouhi N, Griffin S, et al. Associations between exposure to takeaway food outlets, takeaway food consumption, and body weight in Cambridgeshire, UK: population based, cross sectional study. BMJ 2014;348:g1464.

22. Mackenbach JD, Rutter H, Compernolle S, et al. Obesogenic environments: a systematic review of the association between the physical environment and adult weight status, the SPOTLIGHT project. BMC Public Health. 2014;14:233.

23. Minaker LM, Shuh A, Olstad DL, et al. Retail food environments research in Canada: A scoping review. Can J Public Health. 2016;107(0):5344.

24. Ni Mhurchu C, Vandevijvere S, Waterlander W, et al. Monitoring the availability of healthy and unhealthy foods and non-alcoholic beverages in community and consumer retail food environments globally. Obes rev. 2013;14(1):108–19.

25. Papas M, Alberg A, Ewing R, et al. The built environment and obesity. Epidemiol Rev. 2007;29:129-43.

26. Holsten J. Obesity and the community food environment: a systematic review. Public Health Nutr. 2009 Mar;12(3):397-405.

27. Scottish Public Health Network. Obesity Route Map. 1.0 ed: Scottish Government; 2011.

28. Shamseer L, Moher D, Clarke M, et al. PRISMA-P Group. Preferred reporting items for systematic review and meta-analysis protocols (PRISMA-P) 2015: elaboration and explanation. BMJ. 2015;2(349):g7647.

For peer review only - http://bmjopen.bmj.com/site/about/guidelines.xhtml

29. Glanz K. Measuring food environments: a historical perspective. Am J Prev Med 2009; 36 (4S).

30. National Heart, Lung and Blood Institute. Qualitiy Assessment Tool for Observational Cohort and Cross-sectional studies. Systematic Evidence Review from the Blood Pressure Expert Panel, 2011. [Cited 12 October 2017]. https://www.nhlbi.nih.gov/health-pro/guidelines/in-develop/cardiovascular-risk-reduction/tools/cohort.

For peer review only

For peer review only - http://bmjopen.bmj.com/site/about/guidelines.xhtml

Supplementary APPENDIX

Search strategy developed for MEDLINE (OVID)

1. Food environment*.tw
2. Fodscape*.tw
3. Food access.tw
4. Food availability.tw
5. Food affordability.tw
6. Food outlet access.tw
7. Food price.tw
8. Food store*.tw
9. Food outlet*.tw
10. Grocer*.tw
11. Convenience store*.tw
12. Supermarket*.tw
13. Restaurant*.tw
14. Food bank*.tw
15. Online shopping.tw
16. Food shopping.tw
17. Economic access.tw
18. Residential.tw
19. Neighbo*.tw
20. Neighbourhood.tw
21. Urban area*.tw
22. Obes*.tw
23. Obesogenic.tw
24. Overnutrition/
25. Overnutrition.tw
26. Over nutrition.tw
27. Overweight/
28. Body Mass Index/
29. BMI.tw
30. Weight adj2 change.tw.
31. Weight status.tw

For peer review only

32. Weight control.tw
33. Waist circumference.tw
34. OR/1-21
35. OR/22-33
36. 34 AND 35

For peer review only

Preliminary data extraction form

Reviewer identification

Date:

Reference ID:

Name:

Article description

Title:

Authors:

Institution:

Corresponding email:

Type of source (journal article/ grey literature):

Year:

Aims and study design

Aims/objectives:

Study design:

Methodology:

1. Settings

a) Country/city:

b) Urban/rural area:

2. Special characteristics of the population:

a) Age structure:

b) Sex structure:

c) Ethnic features:

d) Educational level:

e) Socioeconomic level:

f) Other characteristics:

For peer review only

3. Study exclusions:

4. Exposure:

a) Food environment definition:

b) Food sources/outlet definition:

c) Data source

d) Year exposure data collected:

e) Type of food environment variable:

f) Food environment variable definition:

g) Methods of data collection

5. Outcomes:

a) Reported outcomes:

b) Outcomes definition:

c) Type of measure (self-reported/measured):

Statistical Analysis

a) Modelling strategy
b) Reported bias
c) Reported confounders

Key findings:

Main findings:

Limitations:

Study limitations:

For peer review only

PRISMA-P (Preferred Reporting Items for Systematic review and Meta-Analysis Protocols) 2015 checklist: recommended items to address in a systematic review protocol*

Section and topic	Item No	Checklist item	
**ADMINISTRATIVE INFORMATION**			
Title:			
Identification	1a	Identify the report as a protocol of a systematic review	1
Update	1b	If the protocol is for an update of a previous systematic review, identify as such	NA
Registration	2	If registered, provide the name of the registry (such as PROSPERO) and registration number	14
Authors:			
Contact	3a	Provide name, institutional affiliation, e-mail address of all protocol authors; provide physical mailing address of corresponding author	1
Contributions	3b	Describe contributions of protocol authors and identify the guarantor of the review	15
Amendments	4	If the protocol represents an amendment of a previously completed or published protocol, identify as such and list changes; otherwise, state plan for documenting important protocol amendments	NA
Support:			
Sources	5a	Indicate sources of financial or other support for the review	15
Sponsor	5b	Provide name for the review funder and/or sponsor	15
Role of sponsor or funder	5c	Describe roles of funder(s), sponsor(s), and/or institution(s), if any, in developing the protocol	NA
**INTRODUCTION**			
Rationale	6	Describe the rationale for the review in the context of what is already known	5-8
Objectives	7	Provide an explicit statement of the question(s) the review will address with reference to participants, interventions, comparators, and outcomes (PICO)	8
**METHODS**			
Eligibility criteria	8	Specify the study characteristics (such as PICO, study design, setting, time frame) and report characteristics (such as years considered, language, publication status) to be used as criteria for eligibility for the review	9-10
Information sources	9	Describe all intended information sources (such as electronic databases, contact with study authors, trial registers or other grey literature sources) with planned dates of coverage	11
Search strategy	10	Present draft of search strategy to be used for at least one electronic database, including planned limits, such that it could be repeated	11
Study records:			
Data management	11a	Describe the mechanism(s) that will be used to manage records and data throughout the review	11

For peer review only

Selection process	11b	State the process that will be used for selecting studies (such as two independent reviewers) through each phase of the review (that is, screening, eligibility and inclusion in meta-analysis)	11-12
Data collection process	11c	Describe planned method of extracting data from reports (such as piloting forms, done independently, in duplicate), any processes for obtaining and confirming data from investigators	12
Data items	12	List and define all variables for which data will be sought (such as PICO items, funding sources), any pre-planned data assumptions and simplifications	12
Outcomes and prioritization	13	List and define all outcomes for which data will be sought, including prioritization of main and additional outcomes, with rationale	13
Risk of bias in individual studies	14	Describe anticipated methods for assessing risk of bias of individual studies, including whether this will be done at the outcome or study level, or both; state how this information will be used in data synthesis	13
Data synthesis	15a	Describe criteria under which study data will be quantitatively synthesised	14
	15b	If data are appropriate for quantitative synthesis, describe planned summary measures, methods of handling data and methods of combining data from studies, including any planned exploration of consistency (such as I^2, Kendall's τ)	NA
	15c	Describe any proposed additional analyses (such as sensitivity or subgroup analyses, meta-regression)	NA
	15d	If quantitative synthesis is not appropriate, describe the type of summary planned	14
Meta-bias(es)	16	Specify any planned assessment of meta-bias(es) (such as publication bias across studies, selective reporting within studies) NA	
Confidence in cumulative evidence	17	Describe how the strength of the body of evidence will be assessed (such as GRADE)	NA

*** It is strongly recommended that this checklist be read in conjunction with the PRISMA-P Explanation and Elaboration (cite when available) for important clarification on the items. Amendments to a review protocol should be tracked and dated. The copyright for PRISMA-P (including checklist) is held by the PRISMA-P Group and is distributed under a Creative Commons Attribution Licence 4.0.**

From: Shamseer L, Moher D, Clarke M, Ghersi D, Liberati A, Petticrew M, Shekelle P, Stewart L, PRISMA-P Group. Preferred reporting items for systematic review and meta-analysis protocols (PRISMA-P) 2015: elaboration and explanation. BMJ. 2015 Jan 2;349(jan02 1):g7647.

For peer review only
